# Targeted drug delivery in neurodegenerative diseases: the role of nanotechnology

**DOI:** 10.3389/fmed.2025.1522223

**Published:** 2025-01-29

**Authors:** Rupal Dhariwal, Mukul Jain, Yaser Rafiq Mir, Abhayveer Singh, Bhavik Jain, Pankaj Kumar, Mohd Tariq, Devvret Verma, Khemraj Deshmukh, Virendra Kumar Yadav, Tabarak Malik

**Affiliations:** ^1^Research and Development Cell, Parul University, Vadodara, India; ^2^Department of Life Sciences, Parul Institute of Applied Sciences, Parul University, Vadodara, India; ^3^Department of Biotechnology, Baba Ghulam Shah Badshah University, Rajouri, India; ^4^Centre for Research Impact and Outcome, Chitkara University Institute of Engineering and Technology, Chitkara University, Rajpura, India; ^5^Chitkara Centre for Research and Development, Chitkara University, Baddi, India; ^6^Department of Environmental Science, Parul Institute of Applied Sciences, Parul University, Vadodara, India; ^7^Department of Biotechnology, Graphic Era (Deemed to be University), Dehradun, India; ^8^Department of Biomedical Engineering, Parul Institute of Technology, Parul University, Vadodara, India; ^9^Marwadi University Research Center, Marwadi University, Rajkot, India; ^10^Department of Biomedical Sciences, Institute of Health, Jimma University, Jimma, Ethiopia; ^11^Division of Research & Development, Lovely Professional University, Phagwara, Punjab, India

**Keywords:** nanotherapeutics, neurodegeneration, targeted delivery, small molecules, Neurodegenerative disease

## Abstract

Neurodegenerative diseases, characterized by progressive neuronal loss and cognitive impairments, pose a significant global health challenge. This study explores the potential of nanotherapeutics as a promising approach to enhance drug delivery across physiological barriers, particularly the blood–brain barrier (BBB) and blood-cerebrospinal fluid barrier (B-CSFB). By employing nanoparticles, this research aims to address critical challenges in the diagnosis and treatment of conditions such as Alzheimer’s, Parkinson’s, and Huntington’s diseases. The multifactorial nature of these disorders necessitates innovative solutions that leverage nanomedicine to improve drug solubility, circulation time, and targeted delivery while minimizing off-target effects. The findings underscore the importance of advancing nanomedicine applications to develop effective therapeutic strategies that can alleviate the burden of neurodegenerative diseases on individuals and healthcare systems.

## 1 Introduction

### 1.1 Neurodegenerative diseases: a growing global burden

Neurodegenerative diseases represent a significant and growing global burden, signified by progressive neuronal loss in the brain or spinal cord, leading to various cognitive and motor impairments (1). These disorders are associated with neuroinflammation, oxidative stress, and neuronal depletion, contributing to conditions including Alzheimer’s disease, Parkinson’s disease, Huntington’s disease, and amyotrophic lateral sclerosis (2, 3). Neurodegenerative diseases pose a growing global burden, impacting millions of individuals worldwide. The multifactorial nature of these disorders, involving genetic, epigenetic, and environmental factors, underscores the complexity of their pathophysiology. Mitochondrial dysfunction is a key factor in the development of neurodegenerative diseases affecting processes like mitophagy and resulting in the buildup of damaged proteins and impaired energy metabolism (4, 5). Additionally, oxidative stress and inflammation are key components of these disorders, contributing to neuronal degeneration and disease progression (6, 7). The disruption of redox signaling and altered protein homeostasis are common features observed in Alzheimer’s, Huntington’s, and Parkinson’s disease (8). Furthermore, genetic and epigenetic factors play crucial roles during the development and advancement of neurodegenerative diseases. Somatic mosaicism, DNA methylation, and non-coding RNAs have has been associated with the underlying mechanisms of disorders like Parkinson’s disease and synucleinopathies, highlighting the complexity of molecular mechanisms underlying these disorders (9, 10). Moreover, the dysregulation of RNA modifications like m6A methylation has been linked to various neurodegenerative diseases, suggesting a potential avenue for therapeutic interventions (11). The identification of biomarkers for early diagnosis and monitoring of neurodegenerative diseases is essential for improving patient outcomes. Blood-based biomarkers, retinal imaging biomarkers, and amyloid PET imaging have shown promise in detecting pathological changes associated with conditions like Alzheimer’s disease and Parkinson’s disease (12, 13). The gut-brain axis has emerged as a significant area of research for analyzing the pathophysiology of neurodegenerative diseases. The two-way communication between the gut and the brain, as seen in conditions like Parkinson’s disease, highlights the importance of considering systemic conditions in the development of these disorders. Moreover, the role of autophagy, mitophagy, and macrophage migration inhibitory factor in neurodegenerative diseases underscores the crucial cellular mechanisms underlying disease pathogenesis and progression (14–16). By elucidating the underlying mechanisms and developing targeted interventions, researchers could aim to alleviate the burden of these debilitating conditions on individuals and healthcare systems.

## 2 Pathological implications associated with neurodegenerative diseases

### 2.1 Pathophysiology of AD: amyloid beta plaques and tau tangles

Alzheimer’s disease (AD) is a neurodegenerative disorder characterized by dementia, including memory loss and impaired cognitive functions like speech, judgment, and abstract thinking, without affecting consciousness (17, 18). As the 7th leading cause of death globally, AD primarily affects aging populations, with 45 million individuals currently diagnosed—a figure projected to reach 74.7 million by 2030 and 131.5 million by 2050. Familial AD (1% of cases) follows an autosomal dominant inheritance involving mutations in *APP*, *PSEN1*, or *PSEN2* genes. Additionally, genetic risk factors like *APOE4* increase susceptibility to late-onset AD, Down syndrome, Lewy body dementia, and vascular dementia (19, 20). Alzheimer’s disease (AD) is characterized by amyloid plaques and neurofibrillary tangles, leading to neuron loss in the neocortex and basal forebrain (21, 22). Amyloid-β (Aβ), a 4kD protein, forms through cleavage of the amyloid precursor protein (AβPP), a transmembrane protein with diverse cellular functions. Cleavage by α-secretase (non-amyloidogenic pathway) produces CTF83 and sAβPPα fragments, while β-secretase (amyloidogenic pathway) produces sAβPPβ and CTF99 fragments. Further cleavage by γ-secretase generates Aβ, primarily Aβ40 and Aβ42, the latter being highly aggregation-prone and toxic (23–25). Aβ monomers aggregate into toxic oligomers via nucleation, which elongate into fibrils. Secondary nucleation and fibril fragmentation amplify Aβ aggregation, resulting in senile plaques rich in Aβ42 due to its faster aggregation (26, 27). Tau tangles, or neurofibrillary tangles (NFTs), are a key pathological hallmark of Alzheimer’s disease (AD). These intracytoplasmic aggregates of tau protein, encoded by the MAPT gene on chromosome 17q21, disrupt normal neuronal function. Tau, essential for microtubule formation, maintenance, and safeguarding DNA and RNA, becomes dysfunctional due to post-translational modifications (e.g., phosphorylation, ubiquitination, and acetylation) and gene mutations (28–30). Abnormal tau phosphorylation reduces its microtubule affinity, promoting aggregation into NFTs and paired helical filaments. Genetic factors, such as mutations in glycogen synthase kinase-3β and CDK5, further drive this process (31). Tau pathology is also influenced by proteins like Aβ, Fyn kinase, Pin1, immunophilins FKBP51/52, heat shock proteins, and α-synuclein (32–34).

### 2.2 Parkinson disease

Parkinson’s disease (PD) is a long-term neurological disorder marked by the progressive decline of dopamine-producing neurons in the substantia nigra area of the brain. The degeneration of dopaminergic neurons in Parkinson’s disease leads to the development of various motor symptoms, including bradykinesia, rigidity, tremors, and postural instability. Non-motor symptoms may also manifest, including depression, sleep disturbances, and cognitive challenges (35). As PD-causing genes can undergo mutations environmental or genetic factors may have contributed to the emergence of PD. Parkinson’s disease (PD) risk can be significantly elevated by conditions such as head trauma or exposure to toxic substances (36). The majority of PD treatments now available target genetic variations or mutations and use pharmaceutical interventions to reduce motor symptoms; still, they do not address the underlying neurodegeneration. One of the primary obstacles in treating Parkinson’s disease (PD) is effectively delivering therapeutic agents to the brain, the blood–brain barrier (BBB) serves as a significant challenge, severely limiting the entry of many drugs into the central nervous system (CNS) (37, 38).

### 2.3 Huntington disease

A CAG repeat expansion due to mutations in the HTT gene encoding huntingtin is responsible for the development of Huntington’s disease (HD), a progressive neurological disorder. It is characterized by a general shrinkage of the brain and degeneration of the striatum (caudate nucleus and putamen), with specific loss of efferent medium spiny neurons (MSNs). HD leads to impairment in proteasome activity as a consequence of the expression of polyglutamine-expanded huntingtin, compromises neuronal homeostasis by decreasing the transcription of essential genes in neurotransmission and signaling and enhances the JNK3 phosphorylation of kinesin heavy chain, which disrupts its binding to microtubules leading to impaired and slow axonal transport. Although both wild-type and expanded huntingtin get cleaved, the presence of mutant fragments correlates with increased toxicity, which might be because of their higher propensity to form nuclear versus cytoplasmic less-toxic aggregates. The discovery of several effective small-molecule therapeutics for HD therapy has revolutionized HD therapy research (39, 40). While there is currently no cure for Huntington’s disease (HD), treatment options are available, including traditional methods and direct cell reprogramming techniques.

### 2.4 Multiple sclerosis

Multiple Sclerosis is a chronic inflammatory disease of the central nervous system (CNS), which leads to large focal lesions in the in the gray matter, including the cortex, the basal ganglia, brain stem, white matter of the brain and spinal cord, characterized by primary demyelination with a variable extent of axonal loss (41). The demyelinating process is associated with activation of astrocytes during the state of active tissue injury and the formation of gliotic scars in inactive lesions. At the sites of active demyelination and tissue injury, microglia and macrophages expresses nicotinamide adenine dinucleotide phosphate (NADPH) oxidase, indicating oxidative tissue damage. Regarding the inflammatory response, CD3-positive T cells are the most numerous lymphocytes in the MS brain. Current MS treatments target disease relapses and aim to reduce further demyelination and disability alongside focusing on management and prevention of acute attacks and lifestyle modifications. The current MS therapy is also associated with low efficacy due to the presence of BBB and the occurrence of side effects due to the dispersion of the drugs that fail to enter the CNS and interacts with non-target cells (42). Therefore, the selective and almost exclusive release of the drug to the action site would lead to considerable therapeutic advantage. Therefore, The use of nanoscale materials could provide more significant therapeutic improvements owing to their improved drug solubility and bioavailability, targeted delivery and controlled release, capability to act as carriers of relevant molecules and trigger an immunomodulatory effect.

### 2.5 Spinal muscular atrophy

5q-Spinal muscular atrophy (SMA) is an inherited autosomal neurodegenerative disease caused by the homozygous deletion of *Survival Motor Neuron-1* (*SMN1*). The different forms based on severity of symptoms in SMA includes, a severe form (type I; Werdnig–Hoffmann disease), an intermediate form (type II) and a less severe disease or ‘juvenile’ form (type III; Kugelberg–Welander disease) (43). SMA type 1 describes infants with disease onset before 6 months of age who do not achieve the milestone of independent sitting. Similarly, SMA type 2 includes children with disease onset typically between 6 to 18 months of age who achieve the ability to sit independently, but do not achieve independent walking. SMA type 3 has individuals ranging from 18 months to adulthood in life span and could walk at some point in their lives. The clinical features of the disease are caused by specific degeneration of α-motor neurons in the spinal cord, leading to muscle weakness, atrophy and, in the majority of cases, premature death (44). Numerous therapies that attempted to treat SMA, such as increasing the number of *SMN2* gene copies with hydroxyurea and increasing the level of full-length *SMN2* mRNA/protein with valproic acid, were carried forward through development in clinical studies (45). However, these are not being pursued further in clinical development due to their insufficient effectiveness.

### 2.6 Amyotrophic lateral sclerosis

In Amyotrophic Lateral Sclerosis (ALS), there is a degeneration of motor neurons located in the brain, brainstem, and anterior spinal cord leading to a gradual loss of muscle function, resulting in progressive paralysis and ultimately, death. In 90% of cases, ALS is considered to have been a sporadic disease; however, 10% of patients show an autosomal dominant (AD) transmission history in their families (46). The pathophysiological mechanisms of the disease include glutamate excitotoxicity, free radical-mediated oxidative stress, structural and functional abnormalities in mitochondria, proteostasis, abnormal RNA metabolism, nucleocytoplasmic transport detects, impaired DNA damage and DNA repair, neuroinflammation, oligodendrocyte dysfunction, and axonal transport defects. Due to impaired glutamate uptake by astrocytes, the neurotransmitter glutamate increases and accumulates in the synaptic cleft, which in turn causes elevated Ca^2+^ influx in MNs (47). Under physiological conditions, mitochondria can remove the increased Ca^2+^ ions, but because of mitochondrial dysfunction, calcium ions accumulate in the cytoplasm and can enter the mitochondria to further exacerbate mitochondrial dysfunction. Moreover, activation of the calcium-dependent enzyme pathway promotes oxidative stress and impairs mitochondrial function. Mutated, misfolded proteins (e.g., SOD1, TDP-43, FUS and C9ORF72) form intracellular aggregates that cause impaired proteostasis and contribute to increased oxidative stress, mitochondrial dysfunction, and axonal transport dysfunction. There are only two drugs approved for the treatment of ALS that includes Riluzole and edaravone (a free radical scavenger) which only extend life by a few months, and also the low stability in biological environments, BBB hinderance, rapid enzymatic degradation, immune system clearance, unfavorable pharmacokinetic properties or inappropriate release profiles imposes a hindrance in therapeutic interventions (48). In the last few years, significant progress has been achieved in the nanotechnology field and could be further used for improving the efficacy of siRNA, ASOs, pDNA like molecules.

## 3 Challenges in diagnosis and treatment of neurodegenerative diseases

### 3.1 Diagnostic odyssey

The diagnostic odyssey for rare neurodegenerative disorders is a lengthy and unpredictable process. Genetic and phenotypic variability in such conditions creates significant challenges for clinicians and uncertainty for patients and families (49). While common medical conditions often result in quicker diagnoses, rare disorders can take months or years to identify. Initial tests may suggest a diagnosis but are rarely definitive, leading to repeated referrals and costly, often invasive, procedures. Despite these efforts, many patients remain undiagnosed or face misdiagnoses (50, 51). This prolonged uncertainty causes frustration, isolation, and distress for families. A prominent example is Amyotrophic Lateral Sclerosis (ALS), a terminal disease characterized by motor neuron degeneration. In a study of 24 ALS patients, 80% received an incorrect initial diagnosis, with only five achieving an accurate diagnosis within 6 months (52). Factors contributing to the diagnostic odyssey include patient-specific issues, clinician expertise, and systemic healthcare challenges.

### 3.2 Clinical and genetic heterogeneity

Neurodegenerative disorders present a growing medical challenge at present due to the complex interplay between genotype and phenotype factors. Neuro-disorders with marked allelic and locus heterogeneity often show individually large, rare, and even private conditions (53). The phenomenon of heterogeneity in rare neurodegenerative disorders can be attributed to many observed features such as different severe and rare mutations in the same gene in unrelated individuals, different phenotypes observed in different individuals, and mutations in different genes in the same or related pathways may cause the same disorder (54). Neurodegenerative disorders, like FTD and AD, show biological heterogeneity, in terms of *in vivo* disease biomarkers, such as protein measurements from lumbar puncture, volumetric measurements from imaging, and behavioral measurements from psychometrics. In AD, 25% of patients have an unusual distribution of neurofibrillary tangles, which is defined as limbic-predominant or hippocampal-sparing (55, 56). As a result, it limits their application for patient classification as well as research purposes (57).

### 3.3 Blood cerebrospinal fluid barrier

The Blood Cerebrospinal Fluid Barrier (B-CSFB) serves as a second physiological and anatomical barrier that drug molecules must navigate after crossing the Blood–Brain Barrier (BBB) to reach the brain. B-CSFB is localized exclusively within the choroid plexus (CP) of the brain’s ventricles and is composed of tight junctions of CP epithelial cells, arachnoid membrane, and periventricular organs like pineal gland, median eminence, area postrema. CP secretes and separates cerebrospinal fluid (CSF) from blood and also lines the subarachnoid space around the brain (58). B-CSFB comprises of many transporters like K^+^ channels, glucose transporter-1, Na^+^-K^+^-ATPase and aquaporin, Na^+^-HCO^3−^ cotransporter, Na^+^/H^+^ exchanger, K^+^-Cl^−^ cotransporter involved in the influx and efflux of endogenous substances, and drug molecules to modulate the CSF composition (59–61). It also protects the brain against harmful compounds while providing access to the substances essential for brain functioning; nutrients and hormones, filters the blood contents before reaching the central nervous system, and also maintains the balance of inorganic ions that regulate the activity of neurons (62). Transthyretin and megalin receptors on the CP membrane contribute to the removal of β-amyloid from the brain by transporting it from the CSF via B-CFB, but in AD mice and humans, both proteins show reduced expression (63, 64). Accumulation of β-amyloid changes morphological and functional properties of CP. It downregulates some proteins like Claudin-1, Claudin −5 and occludin which changes B-CSFB barrier in the initial stages of the AD (65). There is also an indication that CP can transport alpha-synuclein between the blood and CSF (66). Similarly, changes in CP are associated with other disorders like multiple sclerosis, ALS and Huntington’s disease. The selective permeability of the B-CSFB acts as a big hurdle in the delivery of brain targeted drugs because they are not allowed to pass through and reach the central nervous system.

### 3.4 Blood brain barrier permeability

Currently, available therapies for neurodegenerative disorders can only control the symptoms rather than treating the root cause of the disease. Failure of the majority of therapies targeted to the brain is at the point of BBB where most of the drugs fail to pass the barrier. BBB is a large complex structure responsible for maintaining a tightly regulated microenvironment inside the brain that blocks substances from passing from the blood into the brain (67). It is made up of the monolayer of endothelial cells, which make up the capillary walls of the brain, joined by tight junctions, which involve cell adhesion molecules like Occludin, Claudin, and other cell adhesion molecules. Other proteins associated with BBB include perivascular macrophages, astrocytes, and pericytes (68). The BBB regulates the active and passive transport-mediated entry of important nutrients and oxygen into the brain and also prevents the entry of neurotoxins, pathogens, and other unwanted substances into the brain (69). The proper operation of BBB is required for maintaining vital functions of the brain because any breach at blood and brain interface can lead to numerous neurological conditions like PD and AD (70). To address this limitations, nanoparticles have emerged as safe and potential molecules to deliver drugs or therapeutic molecules across the BBB (71). Nanoparticles mostly ranging from 1 to 1,000 nm, have the ability to carry high drug concentration, increased drug transmission, low toxicity in the brain along with physical and chemical stability. A variety of natural, synthetic and inorganic nanocarriers such as Polymeric and liposomal particles have been developed for the identifying and managing neurodegenerative diseases (72–74). Inorganic nanoparticles (Gold, Silver, and Cerium) are most exploited for targeted brain drug delivery and imaging applications across BBB due to their size, stability, surface modification, and functional property (75, 76). Various nanoparticles such as Dendrimers, PLGA, 1,2-dimethoxymethano, fullerene, PBCA, Thioflavin-T containing core-shell latex particles and MION for AD, Chitosan, Carbon nanotubes for PD, and Fullerenols, Β-Cyclodextrin, and SLN for Huntington’s disease (71). Nanoparticles present a promising therapeutic option for neurodegenerative disorders across BBB but more investigations are required to overcome a few challenges associated utilizing nanoparticles for targeted drug delivery can address issues such as toxicity and accumulation, and compatibility especially in the brain.

## 4 Nanomedicine: a promising approach for targeted drug delivery

**Figure 1:**
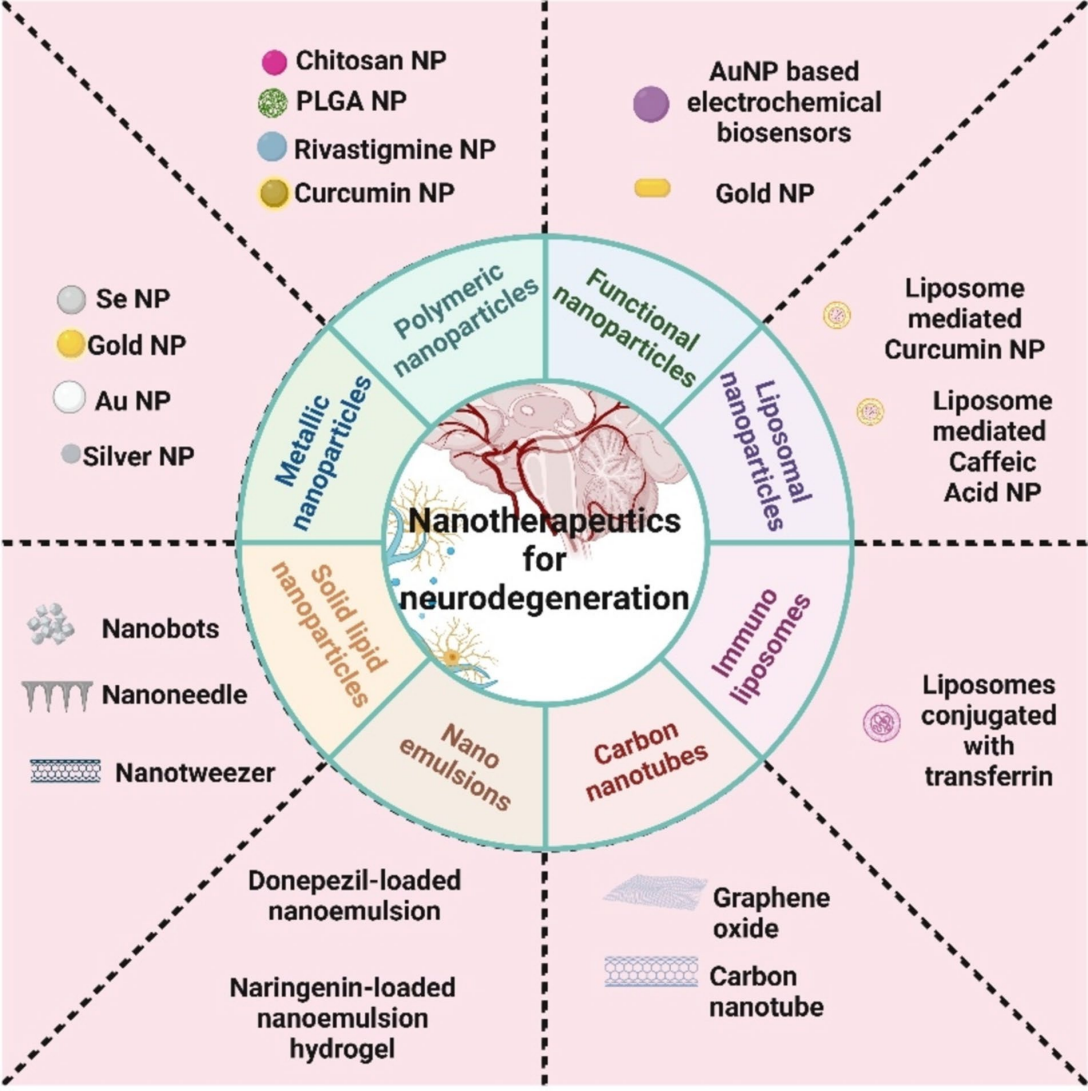
Different classifications of nanoparticles generally used for neurodegenerative disease treatment.

Nanomedicine, a field at the intersection of biomedicine, nanotechnology, and pharmacology, holds great promise for revolutionizing targeted drug delivery (77, 78). By utilizing nanoparticles as drug carriers, nanomedicine aims to enhance drug solubility, prolong drug circulation in the body, and control drug release, thereby improving drug delivery specificity while minimizing off-target effects (79). These drug-containing nanoparticles can be engineered to specifically target tumors using either passive or active targeting mechanisms, leading to improved therapeutic outcomes by reducing drug accumulation in non-target tissues (80). The advantages of nanomedicine include enhanced permeability and retention, extended drug half-life, targeted delivery, and controlled release, even across physiological barriers like the blood–brain barrier (81). However, certain disadvantages associated with the nano therapeutics includes bioaccumulation in the environment, toxicity, high costing for synthesis purpose, etc. Various formulations such as dendrimers, liposomes, drug-polymer conjugates, nanoparticles, and polymeric micelles have been developed as effective vehicles for targeted drug delivery to tumors (82). The different types of nano compositions that are commonly prevailing for neurodegeneration treatment are shown in Figure 1 below.

These nanomedicines interact with the surface of tumor cells, facilitating intracellular signaling that enhances drug accumulation within the cells, thereby improving the efficacy of targeted drug delivery (83). Their applications extend beyond drug delivery to include nanostructures that demonstrate their versatility in medicine through applications such as biosensors, neuro-electronic interfaces, *in vivo* imaging, and cell-specific molecular interactions (84). Nanomedicine formulations that enable precise targeting delivery and intelligent drug regulation hold significant promise for enhancing therapeutic outcomes in various diseases, including cancer (85). Nanoformulations like nanomedicine play a crucial role in improving drug solubility, pharmacokinetics, disease-targeting capabilities, and drug release profiles, ultimately enhancing delivery efficiency (86). The concept of nanomedicine has paved the way for innovative strategies in drug delivery, such as employing nanoparticles in the development of smart and precise drug delivery systems (87). By leveraging the enhanced permeability and retention effect and employing advanced strategies to improve tumor targeting, nanomedicine has demonstrated efficacy in delivering anticancer drugs to tumors with greater precision (88). Nanomedicine has also been explored for combination therapies, integrating chemotherapy and photodynamic therapy to enhance treatment efficacy while minimizing side effects (89). The field of nanomedicine continues to evolve, with ongoing research focusing on improving drug solubility, penetration, and tumor-targeting abilities to enhance therapeutic outcomes (90). The multifaceted applications of nanomedicine in various medical fields underscore its potential to revolutionize drug delivery and improve treatment strategies for a wide range of diseases. The below mentioned Table 1 shows the significance of nanoparticles for neurodegenerative ailments:

**Table 1:** Properties and applications of different types of nanoparticles in neurodegenerative disease therapy.

Sr. no.	Nanoparticle type	Mode of action	Target disease	Reference
1	Graphene quantum dots	Exhibit anti-amyloid activity	AD	(216)
2	Resveratrol-Selenium	Regulates PI3K signaling pathway	AD	(217)
3	Poly(*n*-butylcyanoacrylate)-Rivastigmine	AChE inhibition & improvement in cognition (thinking, memory, speech) and behavioural symptoms	AD	(106)
4	Liposome encapsulated Dopamine	Enhanced Glutathione peroxidase and superoxide dismutase activity thereby, decreasing oxidative stress	PD	(149)
5	Selenium nanoparticles	Reduces neuronal death, relieves from behavioral dysfunction, and, attenuates oxidative stress	HD	(218)
6	Albumin/PLGA nanosystems loaded with dopamine (ALNP-DA)	Effectively crossed the BBB and improved the motor coordination, balance.	PD	(219)

### 4.1 Antibody-based nanoparticles-combined application of antibody fragments and nanoparticles

Over the last few years, nanomedicine has emerged as an exciting area of research that utilizes engineered nanomaterials (NMs) and are used to achieve clinical targets. Nanoparticles have demonstrated an indispensable role for diagnosing and treating various neurodegenerative disorders, including Alzheimer’s disease (AD). There has been a notable surge in the commercial and industrial utilization of nanomaterials. Furthermore, they have been widely utilized in diagnosis, therapy and medical approaches (91, 92). Utilizing nanotechnology in drug delivery systems has enhanced the kinetic profile and bioavailability of pharmaceuticals in biological systems. Nanoparticles have the ability to transport medications through the Blood–Brain Barrier (BBB) due to small sizes, often between 1 nm to 100 nm and serve many advantages like increased specificity, efficacy and stability (93). To date various types of nanoparticles have been employed in the detection and therapy of Alzheimer’s disease (AD), including liposomes, polymeric nanoparticles and magnetic nanoparticles. Monoclonal antibodies have been used to treat AD but most of the studies were terminated after few clinical trials of Gantenerumab Crenezumab, Aducanumab and Solanezumab (94). As of now, no therapies are able to stop the gradual neuronal deterioration associated with AD. Antibody and antibody parts used against amyloid beta (Aβ) aggregates have proved disappointing, as they gets hindered by BBB (67, 95). To successfully navigate the blood–brain barrier and deliver therapeutic molecules into the brain complete mAbs (monoclonal antibodies) have been replaced by conjugation of nanoparticles/antigens to some antibody fragments in the diagnosis and treatment of AD (96). Due to small size antibody fragments does not gets affected by BBB hinderance and can serve as important tools for imaging in addition to its low cast. Nanocarriers offer a great deal in transporting therapeutic antibodies across BBB as they have high tissue penetration, lengthy period of blood circulation, the ability to install ligands, sensitivity to stimuli and controlled toxicity (97, 98). Different types of nanoparticles like gold nanoparticles, polymeric nanoparticles and Superparamagnetic Iron Oxide nanoparticles have been employed for delivery of therapeutic antibody fragments across BBB.

### 4.2 Combining of antibody fragments to gold nanoparticles

Gold nanoparticles (AuNP) present a stable, biocompatible, and functional platform to deliver drugs and other cargo into the brain for treating patients diagnosed with AD and Parkinson’s disease (PD). AuNPs have a property that can be visualized using un labeled non-invasive X-ray computed tomography (CT). Their advantages make them the favorite nano-carriers among all the nanoparticles for transport across BBB (99). Multi-branched gold particles are more useful as brain targeting agents as compared to spherical ones due to their larger surface area which helps in the installation of drugs and is activated by near infra-red light for imaging and photothermal properties (100). AuNP-based intricate structure known as GNRs-APH-scFv (GAS) was designed using gold nanorods (GNRs), thermophilic acylpeptide hydrolase (APH) designated as ST0779, and antibody fragment scFv 12B4. GNRs transform optical energy into hyperthermia which hydrolyzed aggregates of Aβ by absorbing near infrared-light (NIR). The scFv helped in intensifying hypothermia by interacting with Aβ oligomers and fibrils. This GNRs-APH-scFv could find Aβ aggregates and clear excess of it by activating this entire system in the presence of NIR light (101). Using AuNPs and antibody fragments against Aβ_1-42_ fibril demonstrated that it could identify Aβ_1-42_ fibril and assess the severity of AD (102). A study demonstrated the ability of multi branched AuNP as nano vehicles which traverse the BBB without inducing inflammation. The researchers utilized the unique ability of dihydroxyphenylalanine (DOPA) to effectively and selectively cross the blood–brain barrier (BBB) using the large neutral amino acid transporter (LAT)-1 protein. This approach led to the development of L-DOPA-functionalized multi-branched nanoflower-like gold nanoparticles (L-DOPA-AuNF). L-DOPA-AuNF were successfully taken up by macrophages, as observed and do not cause any inflammation *in vitro* human BBB model (hCMEC/D3 monolayers) (103). Thus they present an effective nano particle based vehicle to transport drugs across BBB.

### 4.3 Conjugation of antibody fragments to polymeric nanoparticles

Polymeric nanoparticles range from 1 to 1,000 nm and they may contain active substances that are either surface-adsorbed onto the polymeric core or trapped inside it. Recent years have seen a significant increase in use of polymeric nanoparticles because of their small size (74, 104). Employing polymeric nanoparticles as drug carriers has several benefits, such as the capacity to shield biologically active compounds from the immediate environment, possibility of controlled release and the enhancement of bioavailability and therapeutic index (74, 100, 104). Due to their capacity to carry large therapeutic payloads, polymeric nanoparticles can prove attractive options after intelligent engineering to breach BBB for the treatment of AD and PD. (105) Attempts have been made to synthesize the polymeric nanoparticles of desired size using poly (butyl cyanoacrylate) (PBCA) and polystyrene but the degradation products of these polymers are toxic in nature (106). However, the selective targeting of nanoparticles in the brain is a challenging task. Polymeric nano micelle has recently been used to inhibit Aβ aggregation in the brain parenchyma (107). The researchers utilized a dual pH/redox-responsive polymeric nanomicelle made from poly (ethylene glycol) (PEG) block copolymers, cross-linked with cationic disulfide complexes. This structure was designed to incorporate charge-converted anionic Fabs to regain their charge and bioactivity in acidic conditions. However, when they are in the acidic and reductive environments of endosomes and brain parenchyma, respectively, they gradually disassemble due to ionic interaction loss and disulfide link breakage, which releases a significant amount of bioactive antibodies (107). A study employed smart nano-vehicles (SNVs) linked to pF (ab′)2 as a biosensor, effectively coating the surface of the nanoparticle core. It was found that PF (ab′)2 could detect deposits of amyloid specifically (108).

### 4.4 Superparamagnetic iron oxide nanoparticles

Superparamagnetic nanoparticles (SPIONs) are made up of molecules featuring various compositions that respond to a magnetic field but lose their residual magnetism once the field is no longer present. SPIONs of diameter between 5 and 100 nm range are being tested for many *in vitro* uses as well as clinical works (109). Common methods for preparing these particles include coating core particles with antibodies that target specific cell antigens, allowing for their separation from the surrounding matrix. Common preparation methods for the preparation of SPIONs include coating them with antibodies directed against cell-specific antigens (109). Due to small size SPIONs, resist the BBB hindrance and are thus, used in imaging to improve and increase magnetic resonance imaging (MRI) contrast as they show little toxicity *in vivo* (93). To study the effect of SPIONs regarding the identification and management of AD in the brain of AD transgenic mice Liu et al. (110) developed a system known as W20/XD4-SPIONs. W20 is a scFv that has the potential to identify the oligomers of Aβ specifically. XD4 helped in activation of SR-A scavenger which resulted in phagocytosis of oligomers of Aβ (110). Investigators observed improvement in cognitive abilities and phagocytosis in microglia of mice brain. The study revealed that W20/XD4-SPIONs can be used to diagnose AD early and could be employed to treat AD as it reduced cytotoxicity caused by Aβ oligomers (111).

## 5 Nano-therapeutics for neurodegeneration treatment

### 5.1 Alzheimer’s disease

To date a number of disease-modifying strategies, such as the use of nanoparticles, phytochemicals and small compounds, have been used to treat AD. Out of all these nanoparticles are especially fascinating due to a number of benefits they have. BBB that keeps the brain’s homeostasis intact and shields it from pathogens and toxic substances represent one of the main obstacles to targeting therapies (112). This property of BBB prevents the passage of therapeutic molecules from blood to brain thus posing a challenge in treatment of neurodegenerative disorders like AD. Traditional drug delivery systems utilizing non-lipophilic, high molecular weight molecules are hindered by BBB (113). At present nanoparticles have developed into a promising method to deliver drugs to target Aβ species across BBB because of their small size, they possess a high surface-to-volume ratio, specificity to amyloid aggregates and they can inhibit the formation of fibrils and plaques by inducing conformational changes and hydrophobic interactions (114–117). Based on factors such as shape, size, charge, and surface chemistry nanoparticles can bypass the BBB by active or passive mechanism. Nanoparticles <200 nm are delivered through the process of transcytosis while ultra-small nanoparticles with size <3 nm can traverse the blood–brain barrier through paracellular diffusion.

#### 5.1.1 Strategies for inhibiting Aβ aggregation

**Figure 2:**
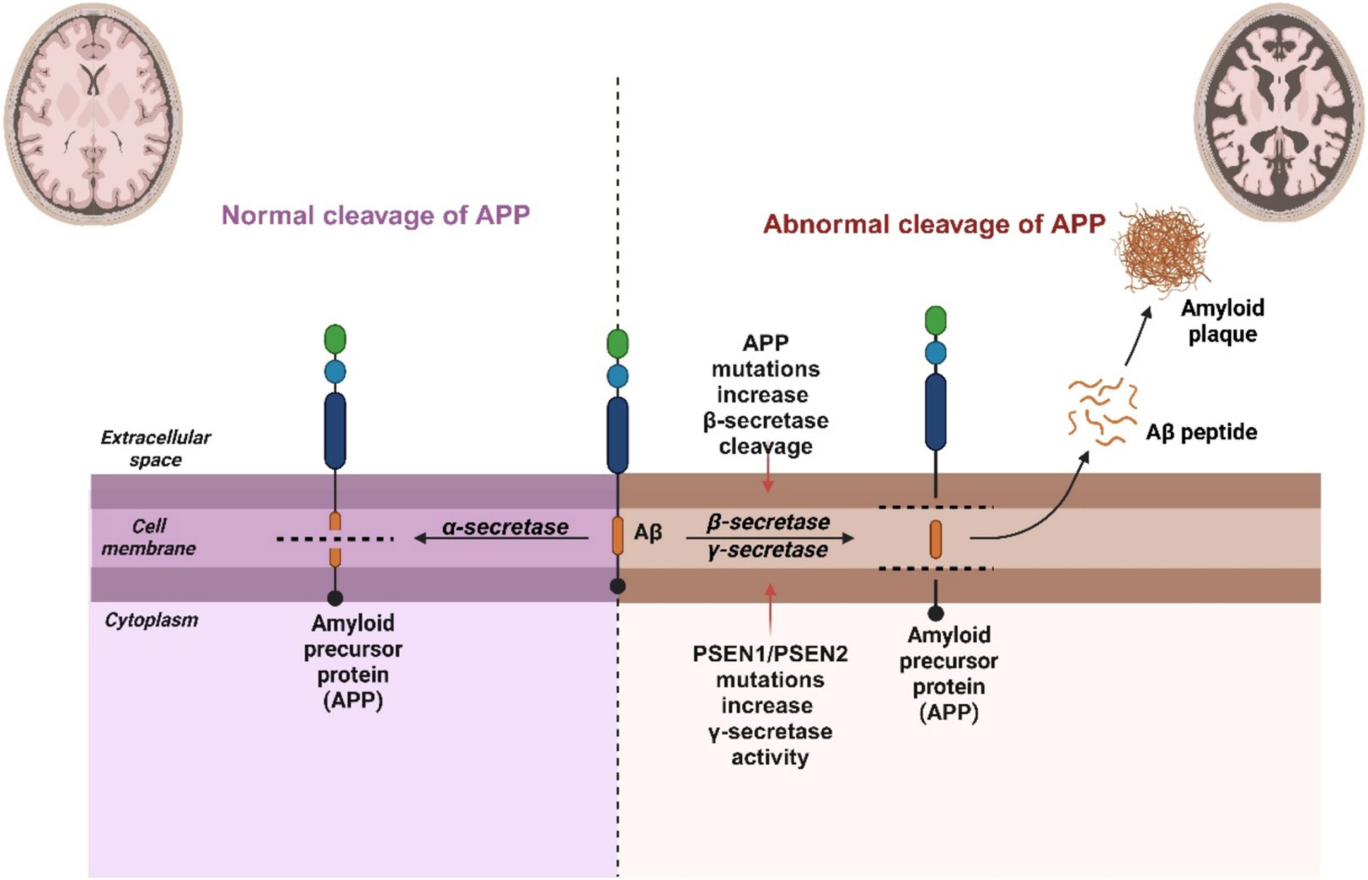
Pathophysiology of amyloid plaques accumulation in a diseased versus healthy individual.

The effectiveness of drugs as well as their capacity to penetrate the blood–brain barrier are key factors in the treatment of Alzheimer’s disease. The general pathophysiology of amyloid plaque generation is further illustrated in Figure 2 above. Nanotechnology-based delivery systems offer a promising solution to address these challenges. Solid lipid nanoparticles (SLNs) represent one of the safest and most cost-effective treatment options available for integrating the blood–brain barrier to treat neurodegenerative illnesses in a non-toxic, safe, and effective manner. Using SLNs can enhance bioavailability without needing high doses (118). They achieve this by bypassing physiological barriers, guiding the active compound to the intended target site, significantly reducing toxicity to nearby tissues, and shielding drugs from chemical and enzymatic degradation. Polymeric nanoparticles (PNPs), commonly used as nanocarriers in biomedical applications, are typically made from biodegradable and biocompatible materials encompass polymers like poly (lactic-co-glycolic acid) (PLGA), polyethylene glycol (PEG), and chitosan, and can be loaded with drugs such as γ-secretase or β-secretase inhibitors (119). The ability of the nanoparticles to target plaques can be improved by surface-modifying them with ligands, like antibodies or peptides, that bind specifically to Aβ. After administering curcumin for 4 weeks using PEG-PLGA nanoparticles linked with Aβ-targeting ligands (120), observed a 40 % reduction in the plaque load (120). In a 2014 study, Balducci showed that phosphatidic acid (PA)-functionalized liposomes may bind to Aβ and prevent its aggregation *in vitro*. These liposomes enhanced cognitive function and decreased Aβ plaque load in a mouse model of AD (121). In a genetically engineered mouse model of Alzheimer’s disease (AD), SLNs loaded with epigallocatechin gallate (EGCG), a polyphenol with anti-amyloidogenic characteristics, managed to penetrate the blood–brain barrier and decrease the deposition of Aβ, according to a study by Ramalho et al. (122). Apart from these types, metallic nanoparticles have also gained significance, particularly in the treatment of neurodegenerative diseases like Alzheimer’s disease (AD). AuNPs coupled with Aβ-specific antibodies have been shown in a study by Liu et al. (123), to target Aβ plaques, promote their disaggregation upon exposure to near-infrared light, and improve cognitive functioning in a transgenic mouse model of AD (123). Researchers have developed other various compounds as well to study the mechanisms underlying the formation and structure of amyloids:

##### 5.1.1.1 Chemical compounds

Antibiotics like Doxycycline Tetracyclines are used to study Aβ (Amyloid-beta)/PrP (Prion protein) /β2-m (Beta-2 microglobulin) /TTR (Transthyretin) structure which depend on the molecular probes that alter their spectroscopic properties when they bind to amyloid fibrils. Fluorescent molecules such as thioflavin-T (ThT) can identify hydrophobic patches of proteins exposed to solvents and can also identify early stages of oligomerization and initial structural changes in amyloidogenic proteins (124). Anthracycline 4-iodo-4-deoxy-doxorubicin (IDOX) has been shown by Merlini et al. (125) to bind with several forms of amyloid fibrils and to prevent native proteins from becoming amyloid. The discovery of IDOX analogs identified tetracycline, which can structurally rearrange the toxic oligomers and reduce the inherent toxicity of these soluble forms (125).

##### 5.1.1.2 Engineered antibiotics and peptides based

Soluble short-sequence beta-sheet breakers influence amyloid aggregate development and stability by binding to amyloidogenic sequences, either preventing fibril formation or promoting the disintegration of pre-existing fibrils (126). Similarly, small designed protein domains like beta-wrapins stabilize amyloidogenic proteins in hairpin conformations, functioning as strong inhibitors of aggregation by blocking self-assembly or disassembling established oligomers (127). Antibodies and fragments such as camel-derived nanobodies also exhibit anti-aggregation properties, preventing the aggregation of amyloidogenic proteins like lysozymes and amyloid-beta. These antibodies are potent diagnostic tools for differentiating amyloid fibrils at various stages of maturation (128). Polyphenols, a diverse group of compounds, are also known for their anti-amyloid and anti-cancer properties. Epigallocatechin gallate (EGCG) inhibits the aggregation of proteins such as alpha-synuclein, polyglutamine-containing proteins, and amyloid-beta by non-covalently interacting with hydrophilic side chains and protein backbones. EGCG also forms covalent bonds with lysine residues via Schiff base formation, enabling irreversible remodeling of proteins into non-toxic aggregates (129, 130). Resveratrol, another polyphenol, delays fibril production and disaggregates preformed fibrils by blocking ring-stacking interactions between specific residues, as evidenced by NMR studies and molecular simulations (131). Additionally, curcumin prevents oligomerization, redirects aggregation towards non-toxic species, and disassembles preformed fibrils of amyloid-beta and alpha-synuclein (132, 133). These approaches highlight the potential of engineered peptides, antibodies, and polyphenols in mitigating amyloid aggregation and related pathologies.

##### 5.1.1.3 Nanoparticles-based targeted destruction

Besides small molecule drugs and protein therapies, nanoparticles have undergone additional research in the past several years as possible amyloid aggregation inhibitors. NPs can target cerebrovascular amyloids, self-assemble an analog of AD proteins, modulate intracellular tight junctions, and penetrate the blood–brain barrier for Alzheimer’s disease treatment (134). Several types of nano-particles have been formulated and designed for effective amyloid-beta degradation. AuNPs have been found to inhibit Aβ fibrillization by binding preferentially to fibrils to form fragmented fibrils and spherical oligomers (135). Similarly, trehalose-based nanoparticles tend to surpass glucose or other sugar-based nanoparticles in terms of reducing protein aggregation, while Carbon nanoparticles derived from sugar are 102 to 105 times more effective than traditional molecular sugars in this area. Exhibiting a hydrodynamic size ranging from 20 to 30 nm, poly (trehalose) nanoparticles engage strongly with the cell membrane via a diverse range of cationic and anionic functional groups. They also promote high endocytotic absorption and effectively prevent protein aggregation (136). Other such effective particles utilize hydrophobic polymer nanoparticles, quantum dots, protein microspheres, carbon nanoparticles, and selenium nanoparticles. Despite some concerns about their potential toxicity, nanoparticles are an expanding field that could pave the way for new therapeutic strategies targeting amyloid-related conditions.

#### 5.1.2 Targeting tau tangles with nanoparticles

**Figure 3:**
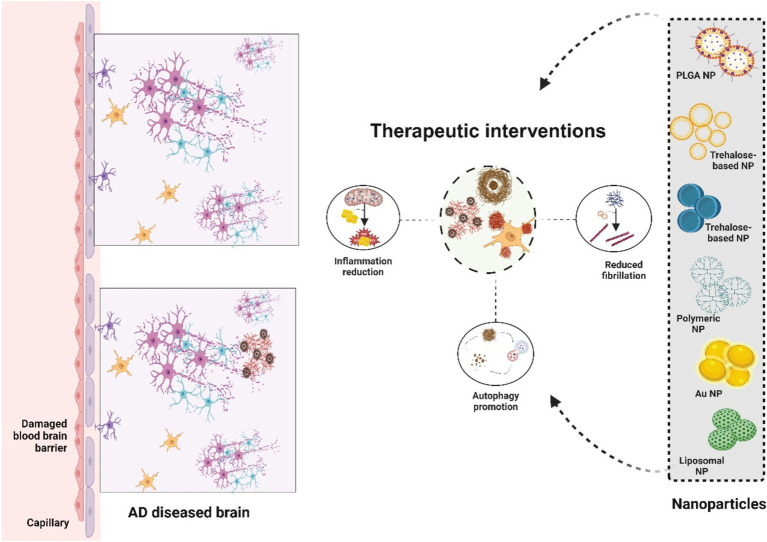
Potential therapeutic strategies for Alzheimer’s disease using nano-therapeutics.

Tau tangles, also known as neurofibrillary tangles, are a hallmark characteristic of Alzheimer’s disease (AD), consisting primarily of hyperphosphorylated tau protein that disrupts normal neuronal function, leading to cell death and the cognitive decline observed in AD patients (137). Targeting tau tangles has developed into a crucial therapeutic strategy in the battle against AD. Alzheimer’s disease is defined by uncontrolled hyperphosphorylation of tau, a microtubule-associated protein that stabilizes microtubules in neurons to maintain appropriate axonal transport and cell structure. This results in the creation of insoluble masses called neurofibrillary tangles. These tau aggregates disrupt neuronal function by destabilizing microtubules and interfering with synaptic transmission, ultimately resulting in neurodegeneration (138). Nanoparticles, which can be developed to carry molecules like peptides, small molecules, or antibodies that specifically bind to tau, offer a promising method for delivering anti-aggregation agents directly to the affected neurons and thereby preventing tau aggregation. The inhibition of tau aggregation is an important therapeutic target. Additionally, these kinase inhibitors can be released from nanoparticles crossing the blood–brain barrier (BBB), blocking the tau phosphorylation-causing enzymes and slowing the initial production of NFTs. Certain chaperone proteins or proteases that naturally degrade tau could be delivered directly to the site of the tangles. Nanoparticles can protect proteins from degradation in the bloodstream and ensure their controlled release within the brain, where they target tau tangles, breaking them down into smaller, non-toxic fragments that can be cleared by the brain’s natural waste-removal processes. Nanoparticles can be loaded with autophagy inducers, such as rapamycin or other mTOR inhibitors, and targeted toward the tau-entangled neurons for clearance. When administered, it activates autophagy by inhibiting the mTOR complex 1 (mTORC1) signaling pathway that normally suppresses autophagy, by the complex formation between the inducers and the neuronal cell surface receptors, thereby, promoting endocytosis (139). Targeting tau tangles with multiple types of nanoparticles has been investigated; tau-binding peptides in combination with gold nanoparticles have been shown to prevent tau aggregation and encourage the breakdown of tau tangles that have already formed (140). Researchers invented lipid-based nanoparticles that contain small interfering RNA (siRNA) that targets tau mRNA. In animal models, these nanoparticles significantly decrease the levels of tau protein by the systematic release of siRNA and degradation of tau mRNA. Also, the presence of lipid coating, facilitate the protection of the siRNA from environmental degradation (141). In cell-based models of Alzheimer’s disease, it has been observed in multiple studies that nanoparticles composed of poly (lactic-co-glycolic acid) (PLGA) that are loaded with a proteasome activator could increase the activity of the proteasome system in neurons, resulting in enhanced degradation of hyperphosphorylated tau and a decrease in tau tangles (142). By delivering drugs directly to the site of tau pathology, nanoparticles minimize the exposure of healthy tissues to the therapeutic agents, thereby reducing the risk of systemic side effects. The figure below (Figure 3) illustrates the therapeutic roles of nanoparticles in the treatment of Alzheimer’s disease.

### 5.2 Parkinson disease

The current medications for PD primarily address motor symptoms and cannot stop the progression of the disease. These treatments mainly focus on replacing DA with drugs such as levodopa (L-DOPA) and DA agonists. These PD treatments face challenges such as rapid degradation, lower bioavailability, decarboxylation before reaching the brain, resulting in a short half-life of approximately 50 min resulting in limited brain penetration (143). Reformulation of drugs in NPs presents a promising solution to address these challenges in PD therapy. Nanotechnology offers a promising solution by improving drug bio-distribution, protecting therapeutic agents from degradation, reducing the necessary drug dosage, and, transporting ligands for enhanced targeted delivery:

#### 5.2.1 Metallic nanoparticles

Gold nanoparticles: Gold-doped TiO nanotubes were designed to detect α-synuclein using photoelectrical approaches successfully. α-synuclein primary antibodies were immobilized on an array plate with subsequent conjugation of secondary antibody-glucose oxidase with gold nanoparticle coating (144). The photoelectric current generation occurred that was proportional to the presence of α-synuclein (145). designed gold nanoparticles for the role of sensing the presence of PD with 78% accuracy by differentiating the breath patterns of diseased and healthy individuals. Similarly, Zhao et al. (146) demonstrated the effect of Nerve Growth Factor-conjugated Au particles on the suppression of α-synuclein aggregates.

Curcumin nanoparticles (147): created nano decoys encapsulated with curcumin nanocrystals that help restore dopamine levels, improve BBB crossing, reduce α-synuclein aggregates, and regulate mitochondrial functioning.

#### 5.2.2 Lipid nanoparticles

Liposomes have the ability to sustain the physiological conditions required for dopamine transport in its baseline state. Thus Jain and Pharma Biotech(148), encapsulated dopamine in liposomes and demonstrated the positive effect on symptomatology in PD rats due to slow, steady, and, prolonged release of DA across the BBB. Further, to enhance the stability of DA within the liposome capsule (149), provided a thiolate CS coating to the complex for reducing the chances of DA autoxidation (150). constructed glutamate-conjugated liposomes for receptor-mediated transcytosis delivery of dopamine.

### 5.3 Huntington’s disease

The current unavailability of treatments for HD has shifted the target toward developing therapeutic strategies using stem cell therapy and nanotechnology, the two promising fields showing potential for treating HD (151). However, stem cell therapy faces several challenges, such as ethical issues, immunological rejection, tumorigenicity, genetic instability, and low survival and differentiation efficiency. Thus, nanostructures have outstanding properties including small size, large surface area, hydrophobicity, strong adsorption capacity, surface modification ability, and, high reactivity:

#### 5.3.1 Polymeric nanoparticles

Koushik (136) have designed a nanoparticle form of trehalose with zwitter ionic surface charge and a trehalose multivalency that showcased more efficiency than molecular trehalose in inhibiting protein fibrillation in extra-cellular space, in blocking aggregation of polyglutamine-containing mutant huntingtin protein in model neuronal cells. This composition worked efficiently even at micromolar concentration compared to millimolar to molar concentrations for molecular trehalose (136).

#### 5.3.2 Metallic nanoparticles

Sarkar et al. (152) explored the efficacy of iron oxide/zinc oxide nanoparticles coated with hemin/amine/arginine/trehalose. It was found that these nanoparticles induce cellular autophagy and increases in cell viability typically up to 7-fold (152).

### 5.4 Multiple sclerosis

Currently, only symptom-based treatments are available that aim at maintaining function and improving quality of life with no FDA-approved therapy available. The application of nanotechnologies is giving rise to entirely new approaches and therapies for targeted drug delivery to various tissues:

#### 5.4.1 Lipid-based delivery systems

A research by Metselaar et al. (220) designed liposomal carriers that could encapsulate water-soluble corticosteroids facilitating long-term circulation, and targeted accumulation at the inflammation site. Similarly, Lomakin et al. (221) invented a nanosystem where the Myelin Basic Protein (MBP) peptide which is highly immunogenic was encapsulated within mannosylated liposomes and thus, could serve as a B cell epitope inactivator thereby, reducing the MS-associated neuroinflammation (153).

#### 5.4.2 Carbon-based nanoparticles

The research by Ionescu et al. (154) implicated carbon nanotubes for diagnostic and stage based MS detection. A cross-reactive array of polycyclic aromatic hydrocarbons and single wall carbon nanotube bilayers could identify the presence of disease in the exhaled breath of patients with multiple sclerosis (154).

#### 5.4.3 Metallic nanoparticles

Research by Ren et al. (155) which has currently advanced towards phase 2 trial laid its focus on the improvisation of energetic metabolisms in CNS cells, supporting neuroprotection and remyelination using CNM-Au8® which is a clean-surfaced gold nanocrystal suspension (155).

### 5.5 Amyotrophic lateral sclerosis

ALS treatment is extremely difficult to implement using conventional strategies, as the limited permeability of the blood–brain barrier (BBB) usually leads to sub-therapeutic concentrations in the brain. Nanotechnology-based strategies, however, employ engineered nanostructures that show great potential in delivering single or combined therapeutic agents to overcome the biological barriers, enhance interaction with targeted sites, improve drug bioavailability/bio-stability, and achieve real-time tracking while minimizing the systemic side effects:

#### 5.5.1 Lipid nanoparticle

Teixeira et al. (156) opted for nanostructured lipid carriers (NLC) that are highly attractive colloidal systems for targeting CNS. The riluzole-loaded lipid nanoparticles showed slow, sustained, less toxicity, and, thus, prolonged riluzole release. These selected optimized lipid nanocarriers (either SLN or NLC) can be exploited as an alternative delivery system to target riluzole to the brain (156).

#### 5.5.2 Graphene mediated delivery systems

Díaz-García et al. (157) explored the efficiency of mesoporous materials as therapeutic agents against ALS by acting as nanocarrier for carrying drug cocktail leptin/pioglitazone (MSN-LEP-PIO). The usage and experiment on diseased mice revealed improved motor coordination, thus, indicating the efficiency of the nanoparticle (157).

#### 5.5.3 Metal nanoparticles

Rzigalinski et al. (158) demonstrated the usage of Cerium nanoparticles for alleviating oxidative stress through its antioxidant activity and also it remains in the brain environment for a longer duration, thereby, exhibiting neuroprotection (158). A similar study by Nabi et al. (159) reported usage of riluzole nanoparticles for improved penetration of drug across the BBB and reduced oxidative stress (159).

## 6 Challenges and future directions of nano-therapeutics

### 6.1 Biocompatibility and safety concerns-Aducanumab and Lacenemab

Development in the field of nanotechnology have developed multiple options of a wide range of nano-medicines for many disorders like neurodegenerative diseases, cancer, and diabetes. Generally, nanomedicines like nucleic acids, proteins, peptides, and other smaller molecules have been utilized for imaging and therapeutic purposes (160). Such nanoparticles have been constructed using; carbon, lipids, silica, silicone, metals, and other polymers (161–163). Lipid, protein, and, polymer-based nanoparticles have been approved for clinical applications (164). However, despite so many developments in nanoparticle-based medicines, they continue to pose unique challenges due to biocompatibility and toxicity issues. Biocompatibility is defined as the capacity of a biomaterial to perform its intended role in relation to a medical therapy, generating the most appropriate beneficial cellular or tissue response in that particular situation and optimizing its relevant clinical performance, without causing any undesired local or systemic change in the recipient of that therapy (165). Toxicity of nanoparticles refers to the adverse effects of nanoparticle therapy on normal structure of tissues/organs and physiological state of recipients (166).

Toxicity of nanoparticles is generally influenced by physiological characteristics such as size, shape, surface charge, as well as chemical properties, and stability (167). It has been observed that nanoparticles like CuO can lead to impaired metabolic activities, mitochondrial and DNA damage which results in reduced cell viability based on their geometry or composition. Due to small size nanoparticles can enter the alveoli of lungs and induce fibrosis, increase ROS and TGF-β production. SiO_2_ was found to induce more bronchiolar epithelial cell damage than TiO_2_. Carbon nanotubes (CNTs) have been described to propogate cancer and fibrosis by activating type 2 immune reaction through the IL1-IL17 and TGF-β axis (168). Size is another factor which decides the safety of nanoparticles. It has been observed that small sized nanoparticles are more pathogenic than larger ones. A study revealed that use of gold nanoparticles having diameter 1.4 nm and capped with triphenylphosphine monosulfonate (TPPMS) lead to cell death, whereas gold nanoparticles of diameter size 3.7 nm and capped with polyethylene glycol (PEG) showed no adverse activity despite their entry into the nucleus of cells (169, 170). It was observed that silver nanoparticles with smaller size (5–10 nm) induced more cytotoxicity than larger ones (15–25 nm) in *Tetrahymena pyriformis* (171). Similarly, smaller size TiO_2_ (20 nm) lead to 43-fold more inflammatory response as compared to TiO_2_ of larger size (250 nm) in a short time study of pulmonary toxicity in rats (172). Similarly, Polymeric nanoparticles (PNPs) despite their potential benefits, toxicity can arise from their physicochemical properties (such as size, charge, surface modifications, and composition), their interactions with cells, and their ability to bypass the blood–brain barrier (BBB). The PNPs can trigger neurotoxicity by interacting with microglia and astrocytes, leading to pro-inflammatory cytokines release. The oxidative stress resulting from this pro-inflammatory storm and ROS generation can lead to damage to cellular structures, including lipids, proteins, and DNA. Polymeric nanoparticles can accumulate in brain tissues over time and might contribute to additional pathological aggregates.

Shape of nanoparticles is also an important factor in deciding its safety and toxicity. Generally, it has been observed that nano rods show more cytotoxicity as compared to spherical ones. An experiment with human lung epithelial cells (A549) disclosed that Zinc oxide (ZnO) nanoparticles that seems rod-shaped exhibits more toxicity as compared to the spherical ones of the same size (173). The use of gold nanoparticles for human dermatocytes revealed that the nanorods of gold coated with hexadecyl cetyl trimethyl ammonium bromide (CTAB) are toxic whereas spherical gold nanoparticles had no natural toxicity (174). Surface charge of nanoparticles is another challenge to the biocompatibility and safety inside human and animal cells/tissues. Toxicity of nanoparticles is greatly dependent upon charge density and polarity. Moreover, different types of cells behave differently with the same nanoparticle. Application of strong positive charged Mesoporous silica nanoparticle to human mesenchymal stem cells and 3T3L1 cell line revealed that former showed good uptake of nanoparticles whereas uptake was inhibited in later (175). Negatively charged particles are strongly uptaken by phagocytic cells as compared to nonphagocytic cells which interact with positively charged nanoparticles. Uptake of cationic nanoparticles by nonphagocytic cells causes plasma membrane disruption which leads to cytotoxicity (176). In another study it was observed that application of positively charged curcumin nanoparticles (coated with polyvinylpyrrolidone) resulted in unstable mitochondria and lysosomes along with apoptosis and ROS production as compared to neutral and negatively charged curcumin nanoparticles (177). Generally, it has been observed that less dense cationic charge produces less cytotoxicity (178). Further non-biodegradable nanoparticles pose another threat to cellular health upon accumulation. Gold nanoparticles have been found in the major groove of DNA and silica particles in nucleoplasm leading to cancer disease (179). Thus size, shape, geometry and surface charge are important factors looked upon to design nanoparticles targeting brain disorders in order to produce safe and biocompatible nano therapies. So it has become important to reveal the molecular mechanism of different interactions between biological systems and nanoparticles in detail to reduce the off-target effects.

There is a need for in-depth analysis of experimental and theoretical models. This will result in deeper understanding of different processes and enhanced biocompatibility of nanoparticles. Aducanomab is a monoclonal antibody with the ability to modify the symptoms of AD. It was prepared by Biogen and the United States Food and Drug Administration (US-FDA) has approved it in June 2021 (180). It was the first nanoparticle based therapy that promised to eliminate the beta-amyloids from the brain and slow down the cognitive and functional deterioration of individuals with early-stage Alzheimer’s disease. It was designed using human B-cell clones that were activated by the neo-epitopes found on the pathogenic Aβ aggregates and this was totally an antibody-based therapeutic approach. Aducanumab bypass the blood–brain barrier (BBB) in preclinical trials, where it was shown to target and remove amyloid plaques from the brains of transgenic mice (181). Clinical trials in three separate studies using 3,482 patients with Alzheimer’s disease revealed magnificent decline of the Aβ-amyloid in dose and time-dependent manner as compared to the control group. Data from these trials supported the FDA’S approval. The safety data from ENGAGE and EMERGE revealed the most frequent adverse effect of Aducanumab in the 10-mg/kg group was Amyloid-Related Imaging Abnormalities (ARIA-E) in 35.2% individuals and 26% patients experienced associated symptoms like headache (47%), dizziness (11%), confusion (15%) and nausea (8%) (182). Biogen has announced discontinuation of Aducanumab in January 2024, with the company claiming a “reprioritazation” of its focus in the AD domain. The possible reasons concerning the discontinuation may include the large size that significantly limits the BBB passage ability, lack of optimal binding affinity for BBB transport receptors, and in long term leads to lesser amount of Abs presence in the CNS. Biogen denied that this decision was motivated by any safety or efficacy worries (183). Lecanemab (BAN2401) is a humanized IgG1 monoclonal antibody designed against Aβ aggregates in patients with early AD and mild cognitive deterioration. Lecanemab is being developed in collaboration between Eisai and Biogen, for curing Alzheimer’s disease (184). It was approved by US-FDA on 6 January 2023 under the Accelerated Approval Pathway. It has the ability to bind effectively to oligomers, protofibrils and other insoluble fibrils. Use of murine version of Lecanemab in animal models has demonstrated reduction in Aβ plaque and Aβ protofibrils (185). Lecanemab is under regulatory evaluation in China, Japan and the European Union and clinical trials are being conducted in a number of other nations (186). However, in clinical trials it was observed that it did not slow cognitive decline in *APOE4* carriers, besides it increased decline in patients with 2 *APOE4* genes (187). The higher levels of amyloid burden in APOE4 homozygotes might lead to a more complex interaction between the drug and amyloid, potentially causing inflammation due to more rapid immune response to amyloid clearance, potentially exacerbating neuronal damage. Also, the dosage of drug provided may not have been sufficient and effective enough for the fast-generating pathology. In spite of so much research in academia and drug industries over hundreds of years we still do not have any reliable and safe treatment for AD. More investment is required in research for neurodegenerative disorders.

### 6.2 *In vivo* efficacy and blood–brain barrier penetration

The blood–brain barrier (BBB) could be considered as an important physiological separator that protects the brain from potentially harmful substances circulating in the blood. It is composed of tight junctions in brain capillary endothelial cells, which restrict the penetration of compounds into the brain (188). The effectiveness of treatments for various brain-related conditions, such as glioblastoma, Alzheimer’s disease, and Parkinson’s disease, is reduced by the hindrance due to BBB (93). The size of nanoparticles hinders their passage through the tight junctions between the epithelial cells of the choroid plexus. Later, the surface charge of particles selectively increases the affinity for binding more strongly to negatively charged components on cell membranes and thereby, increasing their uptake and passage through BBB. Also, the presence of efflux pumps on the Blood-Cerebrospinal Fluid Barrier actively pump the drugs and nanoparticles back into the bloodstream or out of the CSF. The Blood-Cerebrospinal Fluid Barrier also regulates the immunological response occuring due to the activation of complement system that prematurely phagocytose the drug before its therapeutic activation precedes in the system.

The challenge of achieving adequate BBB penetration is particularly significant in the context of treating brain tumors, where the BBB and the blood-tumor barrier (BTB) restrict drug delivery to the tumor site (189). Researchers have been exploring various strategies to enhance BBB penetration for targeted therapeutics distribution in the brain. For instance, the development of engineered extracellular vesicles with specific protein expressions has shown promise in facilitating the delivery of therapeutic agents across the BBB (190). Additionally, the use of cell-penetrating peptides (CPPs) has been investigated to enhance the passage of drugs through the BBB. These peptides possess the ability to facilitate the penetration of nanocarriers loaded with therapeutic cargo, offering a potential solution to the challenge of limited drug passage across the BBB (191). In the realm of nanomedicine, nanoparticles have emerged as promising carriers for enhancing BBB penetration and improving targeted drug distribution to the brain. Studies have demonstrated that certain nanoparticles, such as mesoporous silica nanoparticles functionalized with cell-penetrating peptides, can effectively travel across the BBB and deliver therapeutic agents to the brain (192). Moreover, the innovations in the polymeric nanoparticles tailored for BBB transfer presents a strategic approach to overcoming the drawbacks faced due to the BBB in treating neurological disorders (193).

In the context of specific diseases, such as glioblastoma, researchers have explored innovative drug delivery systems to enhance BBB penetration and improve therapeutic outcomes. Biomimetic drug delivery systems utilizing cancer cell membrane encapsulation have shown potential for targeted drug delivery to glioma sites (194). Furthermore, the combination of immunotherapy and radiotherapy has been investigated as a treatment strategy for brain metastases, with considerations for overcoming the challenges posed by the BBB in delivering therapeutic agents to the brain (195). The efficiency of drug treatments for brain-related conditions heavily relies on the ability of therapeutic agents to pass through the BBB and reach their site of action within the brain. Strategies such as utilizing carrier-free nano-suspensions encapsulated by cancer cell membranes and developing novel peptide-mimicking polymers with BBB-penetrating properties highlight the diverse approaches being explored to enhance drug delivery across the BBB (196–198). By addressing the limitations imposed by the BBB through innovative drug delivery systems and targeted therapies, researchers aim to improve treatment outcomes for various neurological disorders and brain tumors.

The challenge of achieving effective BBB penetration to the brain for targeted drug delivery remains a significant hurdle in the treatment of neurological disorders and brain tumors. Researchers are actively investigating diverse strategies, including the inculcation of nanoparticles, cell-penetrating peptides, and biomimetic drug delivery systems, to enhance BBB penetration and improve therapeutic efficacy. By overcoming the barriers imposed by the BBB, advancements in drug delivery technologies could leverage the treatment options for brain-related conditions, offering new hope for patients facing these challenging diseases.

### 6.3 Clinical translation and regulatory issues

Neurodegenerative diseases, particularly Alzheimer’s disease (AD), pose significant challenges in treatment due to their complex nature and the limitations of conventional therapeutic approaches. In the current years, nanotechnology has developed as a hopeful prospect for developing novel treatments for AD by addressing issues such as poor solubility and targeting efficacy. Nanocarriers, including nano suspensions and quantum dots, offer potential solutions to enhance the treatment of AD by overcoming these challenges (199). The use of nanomedicine in AD management is gaining traction, with various Nano strategies available to develop future therapeutic applications for this chronic neurodegenerative disease (119).

One critical aspect in the translation of nano-therapeutics for neurodegenerative diseases like AD is the development of efficient drug delivery systems. Pure drug nano-assemblies have been proposed as a carrier-free nanoplatform that could streamline the production process and facilitate clinical translation by addressing issues concerned with quality control and production increment (200). However, challenges in clinical translation persist, including issues with reproducibility in manufacturing processes and the need for comprehensive characterization before assessing treatment efficacy (201). In the context of AD, the exploration of microRNA-based therapies has shown potential for addressing the pathophysiological mechanisms of the disease. MicroRNAs play a crucial role in regulating gene expression in AD, and leveraging this knowledge for the development of microRNA-based therapeutics holds promise for future clinical applications (202). Additionally, the use of cell-permeable peptides has been identified as a impactful approach for neurodegenerative diseases cure, including AD, highlighting ongoing efforts to find innovative therapeutic strategies (203).

Furthermore, the role of extracellular vesicles, such as exosomes, in onco-therapeutics has been a subject of interest, showcasing the potential of these vesicles in immune modulation and cargo delivery for therapeutic purposes (204). While the use of extracellular vesicles from mesenchymal stromal cells shows promise for inflammation-related conditions, challenges related to regulatory hurdles and safety precautions need to be highlightes for widespread clinical impact (205). In the realm of nanomedicine, the development of smart nanomaterials for bone regeneration highlights the potential applications of nano-sized vesicles in regenerative medicine (206). Moreover, the utilization of Prussian Blue nanoparticles as a multifunctional agent for various diseases, including neurodegeneration, underscores the versatility of nanotherapeutics in addressing different pathological conditions (207). The usage of near-infrared fluorophores in diagnostics for thrombosis and therapy further demonstrates the diverse clinical potential of nanomedicines in addressing a range of medical challenges (208).

Despite the advancements in nanotechnology for therapeutic purposes, the translation of these innovations from preclinical research to clinical practice remains a significant hurdle. Poor preclinical to clinical translation has been a persistent issue in the development of AD therapeutics, leading to high failure rates in clinical trials (209). The need for improved models, biomarkers, and trial designs is crucial to enhance the success rate of translating preclinical findings into effective clinical interventions (210).

In the context of AD, the generation and aggregation of amyloid-β and hyperphosphorylated tau proteins contributes to neuroinflammation, synaptic dysfunction, and oxidative stress, highlighting the complex pathophysiology of the disease (211). Understanding the roles of non-coding RNA, such as piwi-interacting RNA and microRNAs, in AD pathophysiology provides insights into potential therapeutic targets for addressing the molecular mechanisms underlying neurodegeneration. Additionally, exploring post-translational modifications and biomarkers in AD pathogenesis offers avenues for developing targeted therapeutic interventions (212).

The development of biomarkers and therapeutic approaches for AD is crucial for advancing clinical management strategies. Currently, pharmacokinetic-pharmacodynamic modeling has been advancing for supporting the advancement of the preclinical development and clinical translations that aims to cure and treat tau in AD pathology (213). Furthermore, the translation of blood biomarkers into clinical practice for AD diagnosis presents challenges and perspectives in improving early and accurate disease detection (214). MicroRNAs have also emerged as potential biomarkers for AD diagnosis and therapy, regulating abnormal gene expression related to tau toxicity (215). The field of nanotherapeutics for neurodegenerative diseases, particularly Alzheimer’s disease, is rapidly evolving with promising advancements in drug delivery systems, microRNA-based therapies, and extracellular vesicle applications. However, challenges in clinical translation, regulatory issues, and the complexity of neurodegenerative diseases necessitate further research and collaboration to connect the missing links adjoining the preclinical discoveries and effective clinical involvements.

## 7 Conclusion

Nano-therapeutics provides a broad and targeted set of treatment options for neurodegenerative diseases by addressing the complexities innate in diseases like Alzheimer’s, Parkinson’s, Huntington’s, and amyotrophic lateral sclerosis (ALS). The blood–brain barrier (BBB) and the networking and protecting capability provided by it, pose a challenge for drug delivery, reducing the chances of allowing the passage of therapeutic levels of drugs within the brain with minimum side effects. Nano-therapeutics, however, present innovative solutions by implying nanotechnology to enhance the specificity, bioavailability, and efficacy of treatment options for neurodegenerative conditions. Through innovative nano-carriers, it is becoming easy to deliver drugs across the BBB with enhanced precision, stability, and controlled release. The initiation of multi-functional nanoparticles that allow for both diagnostic and therapeutic capabilities could redefine neurodegenerative disease management, enabling a more proactive approach with real-time monitoring and immediate adjustments to treatment methods.
